# 

**Published:** 1894-09-01

**Authors:** 


**Notices of Births, Deaths, and Marriages** are inserted in this column at a charge of 2s. 6d. for an announcement not exceeding four lines.

**Notice to Advertisers and Subscribers.**

All Advertisements, Orders for Copies of The Hospital, and Business Communications should be addressed to **The Publisher, NOT TO THE EDITOR**, The Hospital, 428, Strand, W.C.

The Cost of Subscription, post free, is as follows:—Per week, 2½d.; per quarter, 2s. 8d.; per half-year, 5s. 3d.; per annum, 10s. 6d. Foreign:—Per Annum, 12s. 8d.; payable, in all cases, in advance.

### NOTICE TO CORRESPONDENTS.

All MS., Letters, Books for Review, and other matters intended for the Editor should be addressed The Editor, The Lodge, Porchester Square, London, W.

The Editor cannot undertake to return rejected MS., even when accompanied by stamped directed envelope.

**"Burdett's Hospital Annual," 1894.**

Fifth year of publication. 900 pp. Boards, gilt lettering. A most complete guide to British, Colonial, Indian, and American Hospitals, Dispensaries, Nursing and Convalescent Institutions, and Asylums. Price 5s. Published by The Scientific Press (Limited), 428, Strand, W.C.

**NOTICE.—The Index for Vol. XV. (ending March 31st, 1894) is on sale at the Office of this Journal, price 2½d., post free.**

### SCRAPS AND GLEANINGS.

Swindon Hospital Saturday collections amounted to £79 3s. 5d. this year.

About £230 was collected on Hospital Saturday at Lewes for the Victoria Hospital.

Three London hospitals have received £10,000 each by the will of the late Mrs. Allan, of Bournemouth.

The Royal Hospital for Incurables at Dublin, now occupied with 182 patients, was lately honoured by a viceregal visit.

Dr. Paton, of Nottingham, has presented £1,000 to the West Kirkby Convalescent Home in memory of his son, recently drowned at Bournemouth.

The Mayor of Cork has given a pair of valuable Sèvres vases as a prize to be offered at the forthcoming fair to be held at Donnybrook in aid of the Eye, Ear, and Throat Hospital.

A serious riot took place lately in the Cossack village of Borgustan in the Caucasus, which arose from popular prejudice against the sanitary precautions imposed by the authorities.

Luton has just seen the inauguration of a new children's home for the town; the new home and its site being the munificent gift of A. P. Welch, J.P., who has already signalised his residence in Luton by many charitable acts.

The third parade of the Durham Friendly Society has recently taken place at the Cathedral, when offerings were taken on behalf of the funds of the Convalescent Home for Friendly Society members established at Grange-on-Sands.

The memorial stone of the new workhouse infirmary in course of erection at the Aston Workhouse, Gravelley Hill, has been laid this month. The new building is to cost £10,000, and will afford infirmary accommodation for 162 patients.

Last week the foundation stone was laid of a crematorium for Liverpool, the crypt of which will be large enough to contain 2,000 urns. The promoters have taken every precaution against the charge of secrecy in disposing of the remains.

Dr. Quintard, of Angers, France, has introduced a new kind of disinfecting stove for the use of surgeons and hospital nurses in destroying germs of disease on surgical instruments. It is made of aluminium, and is hermetically closed.

An appeal is being made for funds towards the improvement and extension of the Heatherdene Convalescent Home at Harrogate. Already some £3,000 has been promised, but a large sum is yet required to complete the necessary amount.

The Borough Fever Hospital at Longshaw, which was opened last month, is now ready for the reception of patients. The building has cost £21,000. It is hoped that there will soon be a convalescent home in connection with the hospital.

A demonstration was held last month at Sunderland in commemoration of the centenary of the infirmary which has just been completed. This celebration took the form of an open air fête, held in the Constabulary Cricket Ground at Hendon.

It is proposed to erect the new general hospital for Kettering and the neighbourhood by voluntary contributions. The greater part of the site is a gift from the Duke of Buccleuch. The work will be proceeded with when sufficient funds have been obtained.

The new Eye Infirmary, presented to Greenock by Mr. Rodger, of Port Glasgow, was formally opened early this month, and handed over to the town. The occasion was signalised by a large trades' demonstration, which paraded the streets on the way to the institution.

Special beds have been prepared for any sudden cases of cholera at the Seamen's Hospital, Greenwich, the Cottage Hospital, Gravesend, and at Guy's Hospital. The Metropolitan Asylums Board have arranged for the immediate removal of suspicious cases to these hospitals.

The St. Pancras Guardians have decided to set up a seaside school for the delicate and scrofulous children committed to their charge. The East Cliff House and grounds at Margate have been purchased for the sum of £8,000, and will forthwith be prepared for the reception of the children.

The Sub-Committee of the Governors of the Bradford Infirmary recently received a large number of friends to tea at the Woodlands Convalescent Home. A band was in attendance and played in the winter garden. Since the opening of the Home in 1877, 11,500 patients have passed through the institution.

The new Dartmouth Cottage Hospital is now opened. The original building was erected in 1887, since when the necessity has arisen for more commodious premises. The committee are anxious that the remaining debt should be cleared off as soon as possible, Mr. Wilkins having meanwhile offered to lend £400 free of interest.

The foundation stone has been laid of the Memorial Cottage Hospital for Crewe. In answer to the appeals from the committee, the sum of £3,391 7s. 5d. has been received and promised, and the site is a gift from the L. and N.W. Railway Company. After the ceremony there was a review of the Boys' Brigade in Queen's Park.

A farm colony for epileptics is being established at Skippings Farm Chalfont St. Peters, by the National Society for the Employment of Epileptics. The institution will accommodate eighteen patients, and arrangements have been made for the immediate employment of the inmates in useful and suitable work on the farm.

The first annual report of the Mirfield Memorial Cottage Hospital has been published this month. The committee stated that the expenditure incurred during the year considerably exceeded the receipts. It is suggested to arrange weekly or monthly collections in aid of the hospital at all the neighbouring factories and workshops.

In commemoration of the founding of the Surrey Convalescent Home for Men, the subscribers received their annual invitation to visit the Home on the last Saturday in July. Great good has been done during the past year by the institution, and new subscriptions are urgently called for. One guinea entitles the donor to an order of admission for two weeks, and the L. B. and S. C. Railway have kindly granted a special travelling fare.

### The Student of Medicine.

### APPOINTMENTS.

C. Eardley-Wilmot, M.D., appointed Senior Assistant Medical Officer to the Middlesex County Asylum, Tooting. H. O. Grenfell, M.R.C.S.E., L.R.C.P.L., appointed Admiralty Surgeon and Agent to Mothecombe, Kingston, and Challaboro; also Certifying Factory Surgeon for Modbury District. C. E. M. Lewis, M.B., C.B.Cantab.. appointed House Surgeon to the West Norfolk and Lynn Hospital, King's Lynn. Richard W. Leslie, M.D., M.Ch.R.U.I., appointed Medical Officer to the Campbell College, Belfast. John S. Maynard, M.B., C.M.Edin., appointed House Surgeon to the Brighton, Hove, and Preston College Hospital and Dispensary. J. P. Molyneux, M.R.C.S.Eng., L.R.C.P.Lond., appointed Police Surgeon for the Haydock, Golboroe, and Ashton-in-Makerfield District. Percy C. Phillips, M.R.C.S., L.R.C.P., appointed Resident Medical Officer to the East London Hospital for Children, Shadwell. L. W. Rolleston, M.B., appointed Junior Assistant Medical Officer to the Middlesex County Asylum, Tooting. A. M. Sheppard, M.B., Ch.M.Syd., appointed House Surgeon to the Central London Ophthalmic Hospital, Gray's Inn Road. H. W. Marett Tims, M.B., M.Ch.Edin., appointed Demonstrator of Anatomy at the Westminster Hospital Medical School.

THE STUDENT OF MEDICINE—*continued.*

### VACANCIES.

The following vacancies are announced this week, the amount of salary in each case being given after the name of the institution:

**Resident Medical Officers.**

General Hospital, Nottingham.—Senior Resident Medical Officer; doubly qualified. Salary £120 for the first year, with an addition of £10 a year up to £150, with board, residence, and washing. Applications to the Secretary by September 1st.

Gravesend Hospital.—House Surgeon. Doubly qualified and registered. Salary £80 per annum, with board and residence. Applications and testimonials to Frederick Mitchell, Honorary Secretary, by September 1st.

Memorial Hospital, Jarrow-on-Tyne.—House Surgeon, age not under 25, and to reside and board free in the hospital. To come under a three years' engagement at a progressive salary of £130, £150, and £170 respectively. Applications and testimonials to J. Campbell, Secretary, by September 4th.

North Wales Asylum, Denbigh.—Second Assistant Medical Officer. Salary £100 per annum, with board, washing, and apartments. Applications to W. Bark, Clerk to the Visitors, by September 12th.

Royal Surrey County Hospital, Guildford.—Assistant House Surgeon. No salary, but board, residence, and laundry found. Applications and testimonials to the Honorary Secretary, by September 17th.

Sheffield Union.—Resident Assistant Medical Officer for the Workhouse at Fir Vale, Pitsmoor. Salary £100 per annum, with apartments, rations, &c. Applications and testimonials before September 5th, to J. Spencer, Clerk to the Guardians.

St. Peter's Hospital, Henrietta Street, Covent Garden, W.C.—House Surgeon for six months. Salary at the rate of 50 guineas a year, with board, lodging, and washing, and an allowance for wine, &c. Applications and testimonials to Irwin H. Beattie, Secretary, before September 1st.

**Non-Resident Medical Officers.**

Cambridge University.—John Lucas Walker Studentship. Applications to Professor Roy, New Museums, Cambridge, by September 30th.

Burgh of Leith.—Medical Officer of Health. Salary to commence at £350. Applications and testimonials to T. B. Laing, Town Clerk's Office, Leith, by September 1st.

General Hospital, Birmingham.—Assistant-Surgeon. Appointment for three years, with eligibility for re-election. Honorarium, £100 per annum. Applications to Howard J. Collins, House-Governor, by September 1st.

Metropolitan Hospital, Kingsland Road, N.E.—Assistant Physician. Must be Fellows or Members of the R.C.P.Lond., and graduates in medicine of a University recognized by the General Medical Council. Applications and testimonials to Charles H. Byers, Secretary, by September 3rd.

St. Bartholomew's Hospital.—Ophthalmic Surgeon, must be F.R.C.S. Eng. Applications and testimonials to W. Cross, Clerk, by September 10th.

### HOUSE APPOINTMENTS.

**St. Thomas's Hospital.**

The following gentlemen have been selected as house officers from Tuesday, September 4th, 1894:—Resident House Physicians: R. E. Nix, B.A., M.B., B.C.Cantab., L.R.C.P., M.R.C.S. (extension); and A. M. Collcutt, B.A., M.B., B.C.Cantab., L.R.C.P., M.R.C.S. (extension). Non-Resident House Physicians: T. G. Nicholson, B.Sc.Lond., L.R.C.P., M.R.C.S.; and A. S. F. Grünbaum, B.A., M.B., B.C.Cantab., L.R.C.P., M.R.C.S. House-Surgeons: H. A. Dickson, L.R.C.P., M.R.C.S.; L. J. Miskin, L.R.C.P., M.R.C.S.; A. W. Cuff, B.A., M.B., B.C.Cantab., L.R.C.P., M.R.C.S.; and W. J. C. Merry, M.B., B.Ch., Oxon. Assistant House-Surgeons: G. J. Arnold, L.R.C.P., M.R.C.S.; and R. Fox Symons, L.R.C.P., M.R.C.S. Obstetric House Physicians—senior, P. C. Fenwick, L.R.C.P., M.R.C.S.; and junior, E. G. E. Arnold, L.R.C.P., M.R.C.S. Clinical Assistants in the special departments for diseases of the throat: F. H. Appleyard, B.A.Cantab., L.R.C.P., M.R.C.S. (extension); and A. F. W. King, L.R.C.P., M.R.C.S. (extension). Clinical Assistants in the special departments for diseases of the skin: A. E. Russell, L.R.C.P., M.R.C.S.; and J. W. Laver, L.R.C.P., M.R.C.S. Clinical Assistants in the special departments for diseases of the ear: S. W. F. Richardson, B.Sc.Lond., L.R.C.P., M.R.C.S.

### Medical Appointments.

THE GENERAL HOSPITAL, BIRMINGHAM.

House Governor.

3rd August, 1894. (637)

ST. PETER'S HOSPITAL, HENRIETTA STREET, COVENT GARDEN, W.C.

